# A novel EEG decoding method for a facial-expression-based BCI system using the combined convolutional neural network and genetic algorithm

**DOI:** 10.3389/fnins.2022.988535

**Published:** 2022-09-13

**Authors:** Rui Li, Di Liu, Zhijun Li, Jinli Liu, Jincao Zhou, Weiping Liu, Bo Liu, Weiping Fu, Ahmad Bala Alhassan

**Affiliations:** ^1^School of Mechanical and Instrumental Engineering, Xi'an University of Technology, Xi'an, China; ^2^Xi'an People's Hospital, Xi'an, China; ^3^Department of Electrical and Information Technology, King Mongkut's University of Technology, Bangkok, Thailand

**Keywords:** brain computer interface, convolutional neural network (CNN), genetic algorithm, EEG, facial expression

## Abstract

Multiple types of brain-control systems have been applied in the field of rehabilitation. As an alternative scheme for balancing user fatigue and the classification accuracy of brain–computer interface (BCI) systems, facial-expression-based brain control technologies have been proposed in the form of novel BCI systems. Unfortunately, existing machine learning algorithms fail to identify the most relevant features of electroencephalogram signals, which further limits the performance of the classifiers. To address this problem, an improved classification method is proposed for facial-expression-based BCI (FE-BCI) systems, using a convolutional neural network (CNN) combined with a genetic algorithm (GA). The CNN was applied to extract features and classify them. The GA was used for hyperparameter selection to extract the most relevant parameters for classification. To validate the superiority of the proposed algorithm used in this study, various experimental performance results were systematically evaluated, and a trained CNN-GA model was constructed to control an intelligent car in real time. The average accuracy across all subjects was 89.21 ± 3.79%, and the highest accuracy was 97.71 ± 2.07%. The superior performance of the proposed algorithm was demonstrated through offline and online experiments. The experimental results demonstrate that our improved FE-BCI system outperforms the traditional methods.

## Introduction

Brain–computer interface (BCI) systems serve as a communication link between humans and peripheral equipment. This technology has been shown to improve the lives of numerous patients suffering from various neurological disorders, including amyotrophic lateral sclerosis and spinal cord injuries (Abiri et al., 2019; Edelman et al., 2019). Over the past few decades, the development of signal acquisition and decoding technology has led to the development of various rehabilitation applications, including neuro-prosthesis (Li et al., 2018a), wheelchairs (Pinheiro et al., 2018), quadcopters (Yan et al., 2020), and robotic arms (Cao et al., 2021).

Several types of brain-controlled systems have been studied; these can be classified into spontaneous and evoked BCI systems (Zhang et al., 2020a). Motor imagery (MI)-based BCI is an important spontaneous BCI system that has been extensively investigated. Reust and his colleagues employed an MI-BCI system corresponding to human hand movement to control two robotic hands; this approach achieved a 95% classification accuracy overall (Reust et al., 2018). Another novel mental imagery system developed by the University of Montreal employed a multimodal BCI system to control the single-step and forward walking status using an immersive virtual reality avatar (Alchalabi et al., 2021).

Numerous BCI studies have focused on evoked BCI systems, such as steady-state visually evoked potentials (SSVEP)- and P300-based BCI systems (Zhang et al., 2021). Zhao et al. demonstrated the feasibility of a new stimulation paradigm that makes full use of peripheral vision, and they used the Manhattan distance for final detection in their research (Zhao et al., 2021). A modified SSVEP-BCI speller with dual-frequency and phase-modulation paradigms was designed at Tsinghua University. It obtained an accuracy of 96% *via* multivariate synchronization index analysis (Yan et al., 2021). P300 BCIs have also been used in a variety of applications for disabled people (Allison et al., 2020; Shukla et al., 2021). A reliable authentication system (based on the P300-BCI system) for protecting against online fraud was designed by Rathi's group. In their study, the optimal performance was observed when using a quadratic discriminant analysis algorithm (Rathi et al., 2021).

To summarize, the merit of spontaneous BCI systems is their stable and rapid responses. However, the long training time and inter-user variability limit further study. Evoked BCI systems achieve high recognition accuracies with low training times; however, this type of BCI system relies entirely upon stimulator design. To solve these obstacles, numerous efforts have been made to develop a novel BCI system in the past few years.

Recently, another type of BCI system based on affective computing was developed. Prof. Lu was the first to report on an emotion-based BCI system; this used a stable electroencephalogram (EEG) decoding algorithm to recognize different emotions (Zheng et al., 2019). Prof. Pan and his colleagues reported a novel facial expression detection method based upon two-decision-level fusion using a sum rule combined production rule (Huang et al., 2017). They subsequently developed a Mindlink-Eumpy software toolbox to classify facial expression information by integrating the EEG signals; this was feasible and efficient (Li et al., 2021). Another representative study was reported by the East China University of Science and Technology, which demonstrated that the presentation of different facial images to subjects could successfully evoke event-related potentials (Jin et al., 2012). In 2018, the present authors used real facial expressions instead of flashing facial images to elicit EEG signals. The experimental results demonstrate the validity of the proposed facial-expression-based BCI (FE-BCI) system (Li et al., 2018b).

Considering all the above, the major challenge in improving the performance of existing BCI systems is the EEG classification accuracy. Most BCI studies have used traditional machine learning or pattern recognition methods to identify relevant information for EEG classification (Zhang et al., 2020b). For example, independent component analysis (ICA) and multivariate empirical mode decomposition (MEMD) are typically used for artifact removal. The wavelet transform (WT) is commonly used for feature extraction and linear discriminant analysis. Back propagation neural network (BPNN)-based classifiers are frequently employed to identify different EEG signals. The EEG decoding method based on spatial information is also widely used in BCI systems to ensure recognition performance. Zhao et al. used combined space–time–frequency features to decode EEG signals. In this study, a deep ConvNet model that combined time-frequency transformations, spatial filtering, and classification was used (Zhao et al., 2019). The University of Glasgow developed a novel space-by-time decomposition method based upon non-negative matrix factorization, to decode single-trial EEG signals (Delis et al., 2016). Nanyang Technological University proposed another space-based EEG decoding method. The time-frequency common spatial pattern method was used to solve the problem of poor classification and robustness in MI-BCI systems (Mishuhina and Jiang, 2021). Jia et al. published one of the most recent studies based on the spatial EEG decoding method, and they employed time-contained spatial filtering to extract spatial and temporal information for EEG multi-classification tasks (Jia et al., 2021).

Unfortunately, these methods are limited by their reliance upon prior experimental knowledge and their low processing capacities for large EEG datasets. These drawbacks also reduce the reliability of BCI systems and further degrade their performance. Following innovations in algorithm development, novel neural network architectures for deep learning have offered the benefits of a smaller reliance upon prior expert knowledge, and automatic feature optimization has recently been employed for decoding EEG signals (Craik et al., 2020). Tang et al. employed a traditional convolutional neural network (CNN) to classify EEG signals from left- and right-hand movements (Tang et al., 2017). Xu et al. used topographically represented energy calculations alongside a novel CNN model to extract time–frequency features from four types of MI tasks. This method improves classification accuracy (Xu et al., 2020). Kwak et al. explored an improved CNN model to distinguish the band power features from different SSVEPs using only two channels (Kwak et al., 2017). Xie et al. combined long short-term memory (LSTM) generative adversarial networks and a multi-output convolutional neural network for MI classification, and their experimental results indicated its favorable performance (Xie et al., 2021). Yu et al. reported a novel adaptive skeleton-based neural network that combined an attentional LSTM network with a 3D convolution, to identify human actions or interactions (Yu et al., 2021b). Moreover, they proposed an LSTM-based network-integrated temporal attention mechanism for spatial human-robot interactions (Yu et al., 2021a). Although these methods can effectively improve the classification accuracy in most MI-BCI systems, the parameters of the CNN model still depend on the researcher's empirical understanding, which leads to poor robustness across different scenarios. Many existing deep learning methods manually set arbitration coefficients or fusion rules according to specific tasks and the researcher's experience.

While GA algorithms have been widely used for CNN parameter optimization in image classification (Sun et al., 2020) or text processing (Liu et al., 2021), the response of EEG signals is entirely different from the 2D image or text information. EEG signals have their unique time-frequency characteristics, and their response from different paradigms is entirely different. So decoding of EEG signals needs a specific architecture of the CNN model and parameter setting guidelines. In particular, although the CNN model-based EEG decoding methods have been studied, only a few works have focused on the FE-BCI system, especially for decoding EEG signals under different expressions.

Despite the number of successful methods available for developing an emotion-based BCI system, it remains challenging to address the dependence of BCI upon the performance of stimulus sources, to thereby ensuring its recognition accuracy. Thus, there remains a need to develop a novel paradigm and expert algorithm that can efficiently identify EEG signals for FE-BCI systems.

The primary objective of this study was to address the dependence of FE-BCI upon the stimulus source and overcome the limitations of long training times and inter-user variability. In this study, an FE-BCI system with four facial expressions (left smirking, right smirking, furrowing brow, and raising brow) was constructed and then used to control an intelligent car. The EEG signals of the proposed FE-BCI system were recorded from the prefrontal and motor cortices. To further optimize the FE-BCI performance, the EEG decoding algorithm constructed using the CNN model combined GA was applied to select the optimal hyperparameter value for the constructed neural network. From our experimental verifications, the main contributions of this work can be summarized as follows:

First, to balance between user fatigue and the classification accuracy of traditional BCI systems, an FE-BCI system identifying four different facial expressions is proposed. It provides an additional option to solve the obstacle between BCI performance and its stimulus reliance. The selected EEG signals from the four different facial expressions are accurately recognized.

Second, to address the issues of the EEG recognition accuracy for different facial expressions, a novel EEG decoding algorithm based upon the CNN model is designed to automatically extract the discriminative features of expression-based EEG signals.

Third, in view of the disadvantages of traditional enumeration methods for hyperparameter value selection, a hyperparameter optimization method based upon the GA algorithm is embedded into the CNN model by setting this model as a fitness function. The CNN model combined with GA is an effective way to optimize the decoding results of EEG signals, further enhancing the overall capabilities of the FE-BCI system.

The remainder of this paper is organized as follows. In Section Materials and methods, the relevant studies and details of the proposed method are presented. The experimental results are analyzed and discussed in Sections Result analysis and Discussion, respectively. The final section concludes this paper.

## Materials and methods

### Related work

With the rapid development of affective computing, emotion recognition has gradually become an important factor when designing natural and friendly human–machine interactions (Svetla and Dimitar, 2015). The mechanisms of emotion states have attracted considerable interest in different research fields (e.g., the physiology, representation, recognition of emotions according to different physiological signals, and their application to affective BCI systems) (Mühl et al., 2014). However, it remains a challenge to distinguish brain responses to different emotional states, owing to spontaneous brain activity (Olderbak et al., 2014). Recent studies have discovered that numerous activities can express emotional states, such as facial expressions, speech, and gestures (Schuller et al., 2005; D'Mello and Graesser, 2009).

Among these factors, facial expressions serve as an effective external feature for depicting emotional states; this has inspired considerable discussion (Wood et al., 2016). Earl et al. reported that brain activity in the prefrontal cortex is related to emotion processing. Friedman and Thayer also demonstrated that changes in facial expressions could produce corresponding brain activity in the prefrontal cortex (Friedman and Thayer, 1991). Moreover, facial expressions are also body movements; thus, they respond to brain activity in the motor cortex (Ross et al., 2016). To summarize, brain activity arising from the prefrontal and motor cortices and attributable to facial expressions can enhance differences when estimating emotion states. Our previous study (Li et al., 2018b) analyzed the mechanisms of facial expressions and further demonstrated that EEG signals from the prefrontal and motor cortices can be discriminated to represent stable emotions. One of the aims of affective neuroscience is to include human emotions in BCI systems. Therefore, facial expressions that represent human emotions can be used in BCI systems. Our previous study also constructed a recognition model based on a traditional machine learning algorithm to distinguish EEG signals arising from different facial expressions; however, the performance of the established FE-BCI system can be further improved. Hence, a new calculation model is proposed in this study. More details regarding the experimental setting and algorithm construction can be found in the following section.

### Subjects and data acquisition

In this study, 16 healthy subjects from 22 to 30 years old (two females and 14 males) participated in the experiment. None of the patients had a history of neurological diseases or any previous experience with the proposed facial expression experiment. Before the experiment, each participant signed a written informed consent form. The Institutional Review Board of the Xi'an University of Technology approved the proposed experiment, and all experiments were conducted in accordance with the Declaration of Helsinki. More details of sample size estimation can be found in Section Statistical analysis.

**Figure 1:**
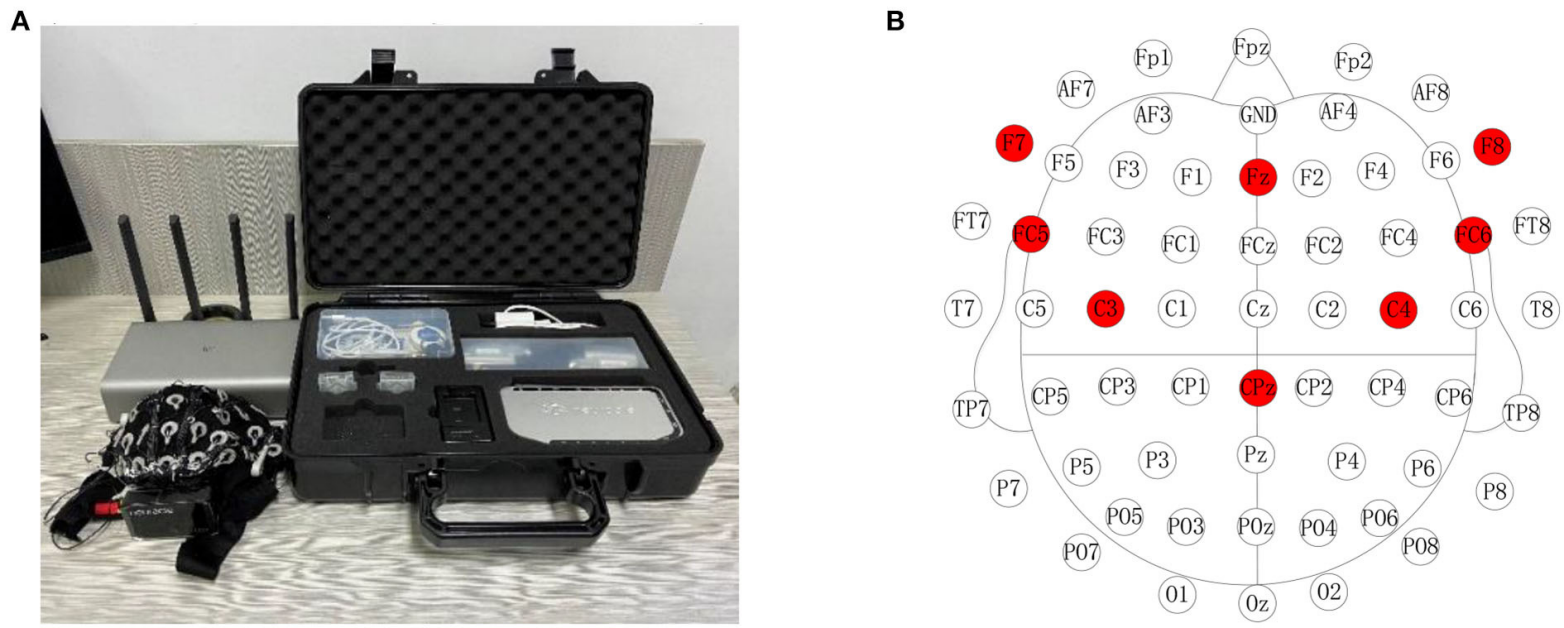
NeuSen-W64 EEG recording system and channel locations. **(A)** NeuSen-W64 EEG recording system. **(B)** Selected eight-channel configuration in NeuSen-W64.

A NeuSen-W64 (Figure 1A) with 64 channels was used to record the EEG signal, and all channel distributions adopted the International Standard 10-20 Electrode Location System. Eight electrodes (FC5, FC6, F7, F8, FZ, C3, C4, and CPz) from the prefrontal and motor cortices were selected to record EEG data. AFz and CPz electrodes were the reference and grounding electrodes, respectively. The electrode distribution and the locations of the selected channels are shown in Figure 1B. During EEG data acquisition, the impedances of all electrodes were maintained below 5 KΩ.

### Experimental procedure

**Figure 2:**
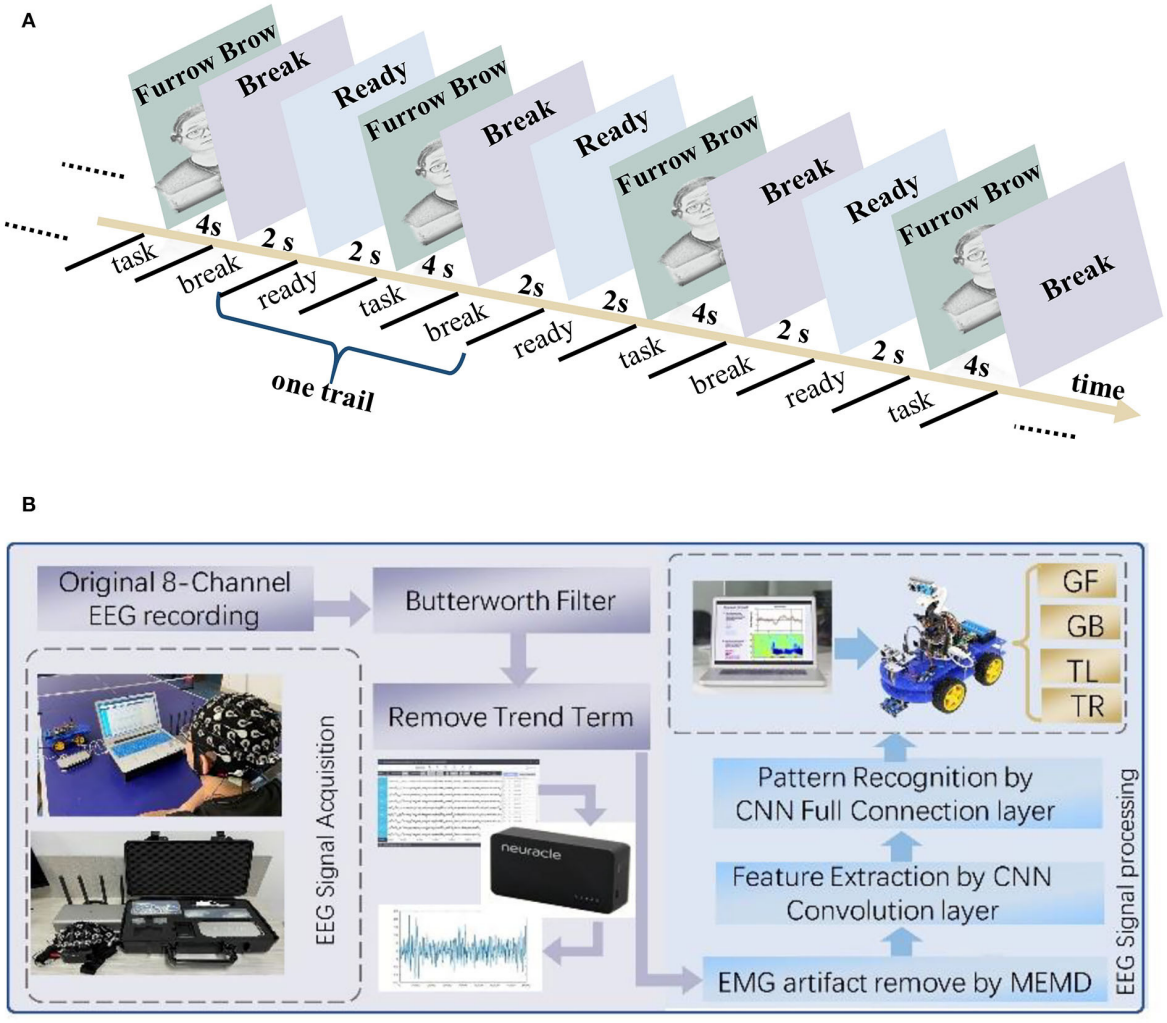
FE-BCI system and its experimental protocol. **(A)** Experimental protocol for the offline experiment. **(B)** Scheme of FE-BCI system for an intelligent car.

According to the facial expression mechanisms, the EEG signals from four facial expressions were collected: furrowing brow, left smirking, right smirking, and raising brow. The subjects were required to keep their bodies stable to prevent noise interference in the EEG signals. The experiment was performed in two steps. The purpose of the offline experiment was to evaluate the efficiency of the proposed CNN-GA and verify the distinguishability of EEG signals under different expressions. The online experiment was to investigate the feasibility of an improved FE-BCI system. For the offline experiment, each facial expression experiment consisted of ten sessions, and each session included six trials. In each trial, the subjects were asked to maintain one of the four selected expressions for 4 s. To avoid mental fatigue, each trial began with a 2 s preparation time and a 2 s rest time when each trial finished. Subjects were allowed a 10-min break when they completed one session. The offline experimental time series is shown in Figure 2A, and the structure of the FE-BCI system is shown in Figure 2B.

The online experiment consisted of six sessions. In each session, the subject was asked to perform one of the four selected facial expressions of their own volition, to control an intelligent car return to the starting position after tracking three targets on four laps: Round 1–4. For the sake of sample balance and its practicality, the start position is distributed in Round 1, Target 1 in Round 4, Target 2 in Round 3, and Target 3 in Round 2. In each session, it contained “change lane to the left” three times, “change lane to the right” three times, “accelerate” five times, and “decelerate” four times. The online experimental process was divided into four stages.

STEP 1: Move the intelligent car from the start position to Target 1. This step includes right-hand lane changes to Rounds 2 and 3, acceleration, deceleration, and another right-hand lane changes to Round 4.

STEP 2: Move the intelligent car from Target 1 to Target 2; this involves acceleration, deceleration, and a left-hand lane change to Round 3.

STEP 3: Move the intelligent car from Target 2 to Target 3; this includes acceleration, deceleration, and a left-hand lane change to Round 2.

STEP 4: Move the intelligent car from Target 3 to the start position; this involves acceleration, deceleration, a left-hand lane change to Round 1, and acceleration to the start position.

The time series for the online experiment matched that of the offline experiment. During the online experiment, left smirking (LS) and right smirking (RS) were used to move the intelligent car 30° to the left and right, respectively. Furrowing brow (FB) and raising brow (RB) were used to produce 0.05 m/s acceleration and deceleration, respectively.

In this study, we used offline EEG data as a training database to construct the improved CNN model, and the online data were used to investigate the generalizability of the proposed method.

### Data analysis

#### Artifact removal algorithm

Brain activity from the scalp is fairly weak: its magnitude is usually in the range of 10–50 μ*V*. Hence, artifacts from the surroundings can easily damage the performance of the BCI system. Depending on the mechanism of signal generation, the artifacts can be classified into power-frequency noise and physiological noise (Mowla et al., 2015).

The Butterworth filter can be applied as an effective linear filter to remove power-frequency noise. Hence, a five-order Butterworth filter with a frequency band of 0.5–45 Hz was initially applied. Subsequently, a noise-assisted MEMD method with highly localized time-frequency representations and self-adaptation was implemented to remove electromyogram (EMG) and electrooculogram (EOG) artifacts (Chen et al., 2018). In this method, the noise-assisted MEMD was employed to decompose the raw EEG signal; then, the sample entropy value of each intrinsic mode function was estimated to detect and remove physiological artifacts. Further details regarding the artifact removal algorithm have been presented in our previous study (Li et al., 2018b).

#### Convolutional neural network algorithm

Deep learning was first introduced by Hinton and Salakhutdinov (2006); it consists of a sequence of convolutions and subsampling layers, in contrast to traditional artificial neural network methods (Craik et al., 2020). CNNs are a representative deep learning algorithm; they offer faster network training, superior conservation of information throughout the hierarchical process, and prevention of overfitting in the built network. These benefits allow the CNN classifier to automatically learn the appropriate features from the EEG data while maintaining its translation invariance and data hierarchy (Xiao and Fang, 2021).

The CNN model in this study consists of several layers, such as convolutional, pooling, dropout, and batch normalization, as well as a fully connected layer. When designing the CNN, the size of the input data and its output results should be taken into consideration. In our study, the input matrix fed into the CNN was 8 × 4,000, where the row corresponds to the eight selected EEG channels and the column indicates the sampling point of 4 s. Because the CNN was used to discriminate the EEG data from four different facial expression tasks, the output layer was designed to have four outputs.

The second component of CNNs is the convolutional layer, which is crucial in facilitating automatic feature learning. In this study, three 2D convolutional layers were designed to perform advanced EEG feature extraction. In each convolutional layer, a convolutional filter whose width matched the dimensions of the input data and whose kernel size of 3 × 3 was applied, to extract the correlation of EEG signals in the adjacent channel and preserve its spatial information. *Via* the convolution of each layer, a two-dimensional feature mapping (combining enhanced information regarding the original EEG data from different facial expression tasks) was acquired.

An important hyperparameter in the convolution layer is the number of kernels, which can sizably reduce the number of weight parameters. To solve the problem of under-fitting (i.e., a small number of convolution kernels) and over-fitting (i.e., a redundancy of convolution kernels), the number of kernel convolutions was adaptively optimized using a GA. More details on the GA can be found in Section Genetic algorithm for hyperparameter optimization.

The pooling layer was inserted after the convolutional layer, to receive the compression feature map matrix from all selected channels and temporal values (Kwon and Im, 2021). The objective of the pooling layer is to improve the statistical efficiency of the network and improve its invariance (and subsequently its robustness). To further reduce the size of the feature map and the number of network parameters, a max pooling layer was used to downsample the feature map and store important information, using a receptive field window size of 2 × 2.

Subsequently, three fully connected layers followed by pooling layers were used to connect all advanced features and then classify them. The first fully connected layer receives a one-dimensional feature vector and outputs the weighted sum of the features to the second fully connected layer. The number of output neurons in the third layer matched the number of facial expression categories to be classified.

**Figure 3:**
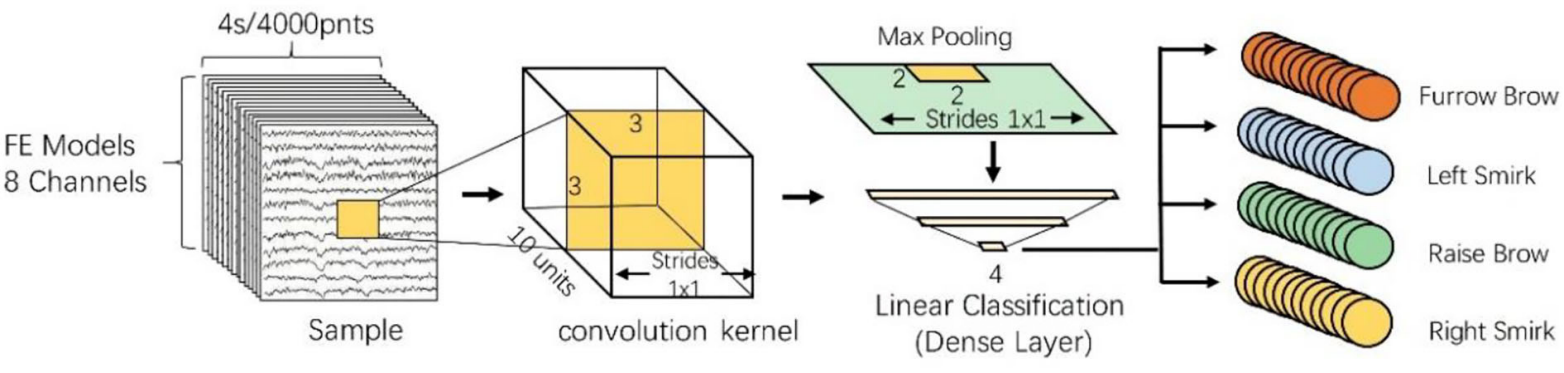
Architecture of the proposed CNN model.

Considering the calculation speed, risk of overfitting, and unsaturated and sparse datasets, the drop-out technique was applied to the fully connected layer, and rectilinear linear unit (ReLU) and Softmax activation functions were applied to each layer, to improve the performance of the proposed CNN models (Stieger et al., 2021). The architecture of the proposed CNN model is illustrated in Figure 3.

As in traditional CNN models, the hyperparameter settings (e.g., the learning rate, batch size, and number of neurons) significantly influences the CNN model performance. Appropriate hyperparameter selections can optimize the performance of the neural network model and further resolve the overfitting problem. Hence, in this study, the GA optimization method was implemented in the constructed CNN model. Two hyper-parameters describing the number of convolution kernels and neurons in the fully connected layer were dynamically optimized *via* the GA evolutionary process. The remaining value of hyperparameters for CNN was the batch size of 16, the learning rate of 0.001, the number of iterations of 100, and the loss function was a cross-entropy loss function.

#### Genetic algorithm for hyperparameter optimization

The hyperparameter optimization of neural networks is a persistent issue. When a neural network is constructed, the key to achieving an efficient model performance is adjusting the hyperparameters, because the performance is highly sensitive to these parameters. When the model complexity increases, the number of hyperparameters increases, and the combination of hyperparameters increases accordingly. It is difficult to determine the exact optimal values of the neural network hyperparameters. At present, mainstream hyperparameter optimization methods include grid search, Bayesian optimization, evolutionary computation, and neural architecture search.

The main advantage of GA is its excellent global search ability, which can quickly search out the whole solution in the solution space without any prior knowledge of the system. Moreover, its characteristic of paralleling process conducts a variety of routes to find optimal results that avoid falling into the fast-falling trap of the optimal local solution. Most important, the superior performance of the GA method is its social ability, which makes it easier to link with other algorithms (Chang and Yang, 2019).

**Figure 4:**
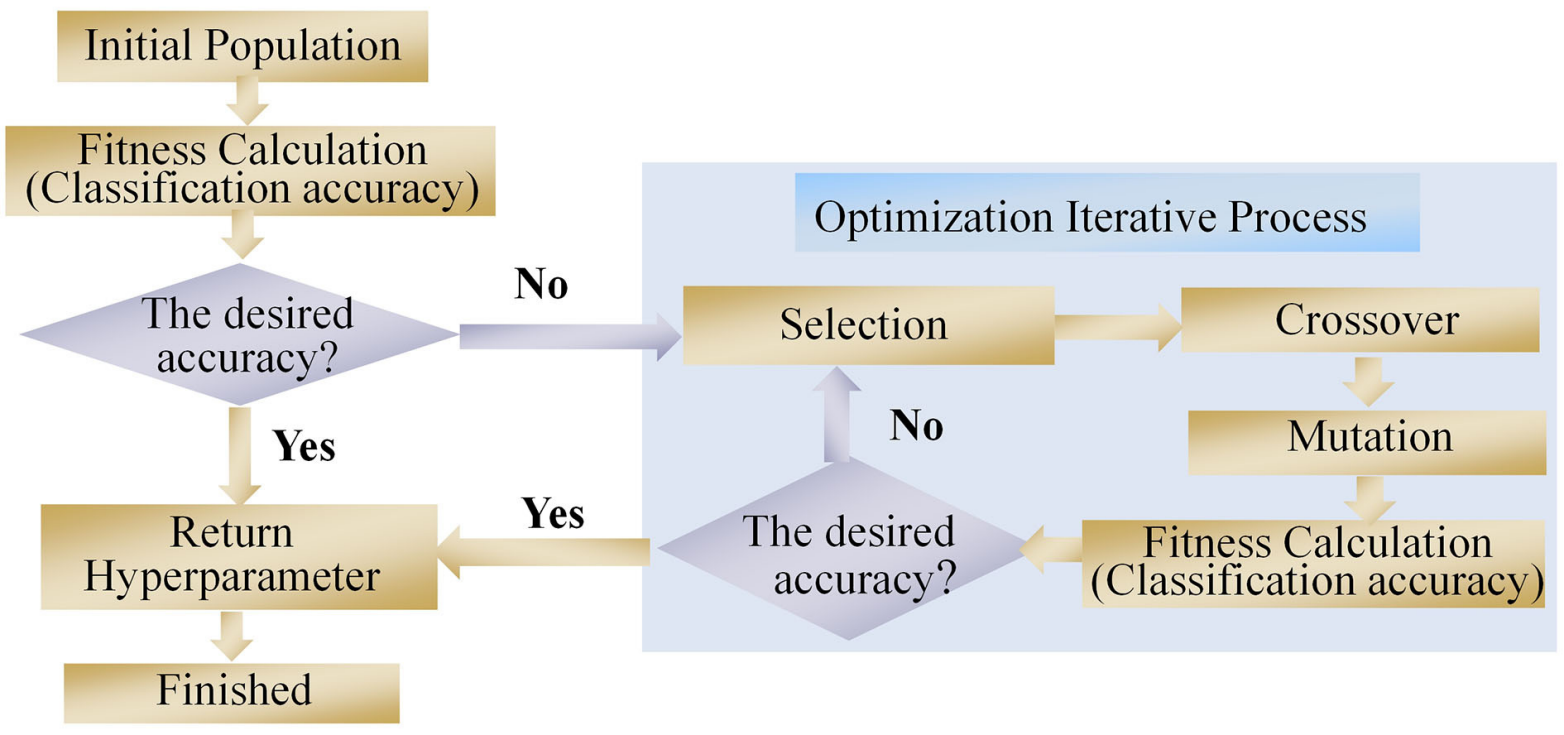
Architecture of the proposed GA.

In this study, a neural network hyperparameter optimization method based on a GA was proposed. GA was first introduced by Holland (2000). It was inspired by the Darwinian theory of survival and the fitness mechanism in nature (Rui et al., 2019). A GA method is a population-based search algorithm whereby each individual in a population represents a set of hyperparameter solutions. Each individual is a set of genes, where each gene represents a hyperparameter. Different gene combinations determine the fitness value of the neural network (i.e., the classification accuracy of the CNN model). The fitness value also determines which individual can transmit its genes to their offspring (i.e., the value of the hyperparameter). A schematic of the GA is shown in Figure 4.

After the initial population is generated, the fitness of each individual is calculated, and the relationship between the fitness and hyperparameters is established. In this study, the hyperparameters describing the number of convolution kernels and neurons in the fully connected layer were encoded via a binary code. The encoding precision δ can be calculated as


(1) $$\begin{array}{r} \delta =\frac{u_{\max}-u_{\min}}{2^{l}-1} \end{array}$$

where *l* is the encoding length, and *u*_*max*_ and *u*_*min*_ are the upper and lower limits of the set hyperparameters, respectively.

The fitness of individuals indicates the applicability of the hyperparameter solutions to the model performance, and superior individuals can be obtained by selecting, crossing, and mutating three genetic operators. In the present study, the roulette method was used to select individuals. The probability P(*x*_*i*_) of each individual is represented as


(2) $$\begin{array}{r} P\left(x_{i}\right)=\frac{f\left(x_{i}\right)}{\overset{N}{\underset{j=1}{\sum }}f\left(x_{j}\right)} \end{array}$$

where *N* is the population size, *x*_*i*_ is the *i*_*th*_ individual, and *f(x*_*i*_*)* is the fitness of the *i*_*th*_ individual.

Crossover operators are generated by two new individuals that exchange gene components between two chromosomes in a certain way. In this study, a multipoint crossover operator was used to pair individuals in the population. The mutation operator is an auxiliary method for generating new individuals; it determines the local search ability of the GA and maintains population diversity.

**Figure 5:**
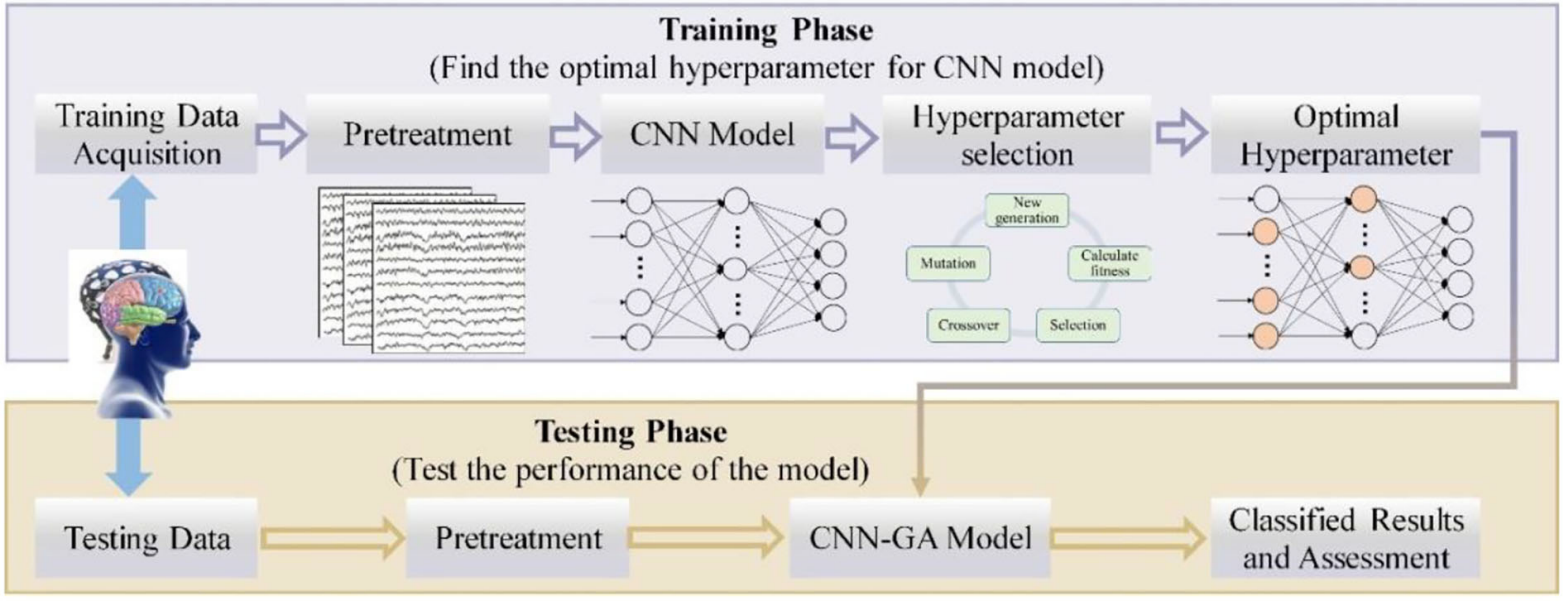
Scheme of the proposed CNN–GA algorithm.

The entire process of the combined CNN–GA method is shown in Figure 5. The EEG signals were recorded from eight channels in the prefrontal and motor regions; hence, the input data for the CNN model were 8 × 4,000, where the rows denote channels and the columns are sampling points. The CNN model employed in our study consisted of three convolutional layers, one pooling layer, and three fully connected layers. Two batch normalization (BN) and one dropout were also used in the proposed CNN–GA model. Because the third fully connected layer is used to output the discrimination result from the four expression-based EEG signals, the number of neurons in this layer was four, and its activation function was selected as Softmax. The initial values for the CNN model parameters were a batch size of 16, a learning rate of 0.001, 100 iterations, and a cross-entropy loss function. The numbers of convolution kernels (in the three convolutional layers) and neurons (in the two fully connected layers) were set *via* GA optimization.

In this study, we designed the proposed CNN model as the fitness function. The numbers of convolution kernels and neurons in the fully connected layer were set as the hyperparameters to be optimized, and the classification accuracy was set as the fitness value in the GA. For the GA algorithm, an excessively large population will increase the time cost. However, too small a population will mean that the algorithm is likely to fall into a locally optimal solution. Based on the relevant literature (Katoch et al., 2020) and experimental analysis, the population size was set as 20 in this study. Because the accuracy of the EEG signals tended to stabilize after 20 iterations, the number of GA iterations was set to 20. The ranges of the five hyperparameters were *u*_*min*_ = {1, 1, 1, 1, 1} to *u*_*max*_ = {20, 20, 20, 512, 512}. Because the hyperparameters in this study were integers, the precision of their values was defined as {1, 1, 1, 1, 1}. The initial values of the hyperparameters were chosen automatically within these ranges. Due to the individual variability, each subject had its own hyperparameter value after optimation.

After optimizing the CNN hyperparameters *via* training data, the testing data were applied to evaluate the model performance using the subject's own classifier. To evaluate the performance of the proposed CNN–GA algorithm, five-fold cross-validation was used to estimate its recognition accuracy. This cross-validation was repeated four times. In each validation, four data subsets were used for training and one was used for testing. Five-fold cross-validation means that 240 offline samples are randomly divided into five equally sized subsets. Four subsets (240/5 × 4 = 192 samples) were used for training the CNN–GA model, and the remaining subset (240/5 = 48 samples) was used to verify the performance of the trained model.

To further evaluate the feasibility of the proposed CNN–GA method, the traditional combined WT–BPNN method and a traditional CNN model were used as comparison algorithms. The WT decomposition level was set to 5, and the db-3 wavelet served as the WT basis function. The energy and variances of the wavelet coefficients were employed as the feature sets of expression-based EEG signals. The three-layer BPNN model (with one hidden layer) was constructed in a previous study. Because the BPNN inputted two WT coefficients from eight channels of each trial, the corresponding input layer of the BPNN had 16 nodes. The output layer had two nodes (to flag the results), and the hidden layer had 20 nodes. Apart from the hyperparameters (that needed to be optimized), the structure of the comparison CNN model and its remaining parameter values were consistent with those of the CNN–GA model. That is, the batch size was 16, the learning rate was 0.001, the number of iterations was 100, and the loss function was a cross-entropy loss function. According to previous studies regarding parameters selection (Craik et al., 2020), the number of convolution kernels in the three convolutional layers was set as 3, and the numbers of neurons in the two fully connected layers were 64 and 32, respectively.

Furthermore, the kappa value serves as a well-known evaluation index for investigating the performance of EEG classification algorithms; it expresses the agreement between the classification accuracy of *p*_0_ and the expected consistency rate *p*_*e*_ for the same categories (Chicco et al., 2021). The kappa coefficient can be interpreted as an agreement measure to determine whether different categories are consistent with their prediction results. The kappa coefficient ranges between 0 and 1, where 0 is consistent (owing to randomness) and 1 is perfectly consistent. The coefficient is defined as


(3) $$\begin{array}{r} K=\frac{p_{0}-p_{e}}{1-p_{e}} \end{array}$$

where *p*_0_ is the classification accuracy and *p*_*e*_ indicates the expected consistency rate.

The formula for calculating the classification accuracy *p*_0_ is defined as


(4) $$\begin{array}{r} p_{0}=\frac{TP+TN}{TP+TN+FP+FN} \end{array}$$

where TP is a true positive, FN is a false negative, FP is a false positive, and TN is a true negative.

*P*_*e*_ is the expected agreement rate, which is the consistency rate attributable to chance. *P*_*e*_ is the accuracy under statistically independent observers, which can be computed *via*


(5) $$\begin{array}{r} p_{e}=\frac{\left(TP+FN\right)\times \left(TN+FN\right)+\left(TN+FP\right)\times \left(TP+FP\right)}{N^{2}} \end{array}$$

where *N* is the number of samples in the dataset.

#### Statistical analysis

In this study, the difference in classification accuracies between the three EEG decoding methods (CNN, CNN–GA, and combined WT–BPNN) was assessed using a Student's paired *t*-test and one-way analysis of variance (ANOVA), respectively. Based on the statistical theory, three parameters of significance level α, the expected effect size *f* , and the desired statistical power (1-β*)* determined the choice of the sample size and verified the significant differences among different methods (Desu and Raghavarao, 1990). The desired effect size was 0.9 (*f* = 0.9), significance threshold was set as 0.05 (*a* = 0.05) and desired statistical power (1-β*)* was 0.8. Furthermore, the Greenhouse–Geisser correction was applied for *p*-value adjustment. Using the statistical software G^*^Power of the given parameters setting and referring to some existing studies (Zheng et al., 2019; Shajil et al., 2020; Cao et al., 2021), the sample size is 16 subjects in this study.

Student's paired *t*-test is primarily used to test whether the same group of subjects differs significantly under two different conditions. We investigated the variability (for the same subjects) between the CNN and CNN–GA algorithms and between the CNN–GA and WT–BPNN methods. Because the *t*-test is only suitable for testing the variability between two conditions, ANOVA was used to investigate the significant differences when more than two conditions differed. Therefore, this method verifies the variability between the CNN, CNN–GA, and WT–BPNN algorithms.

## Result analysis

In this section, the offline and online experimental results are presented. The main purpose of the offline experiment was to evaluate the efficiency of the proposed CNN–GA, whereas the online experiment was performed to investigate the feasibility of the improved FE–BCI system.

### Offline experimental results

**Figure 6:**
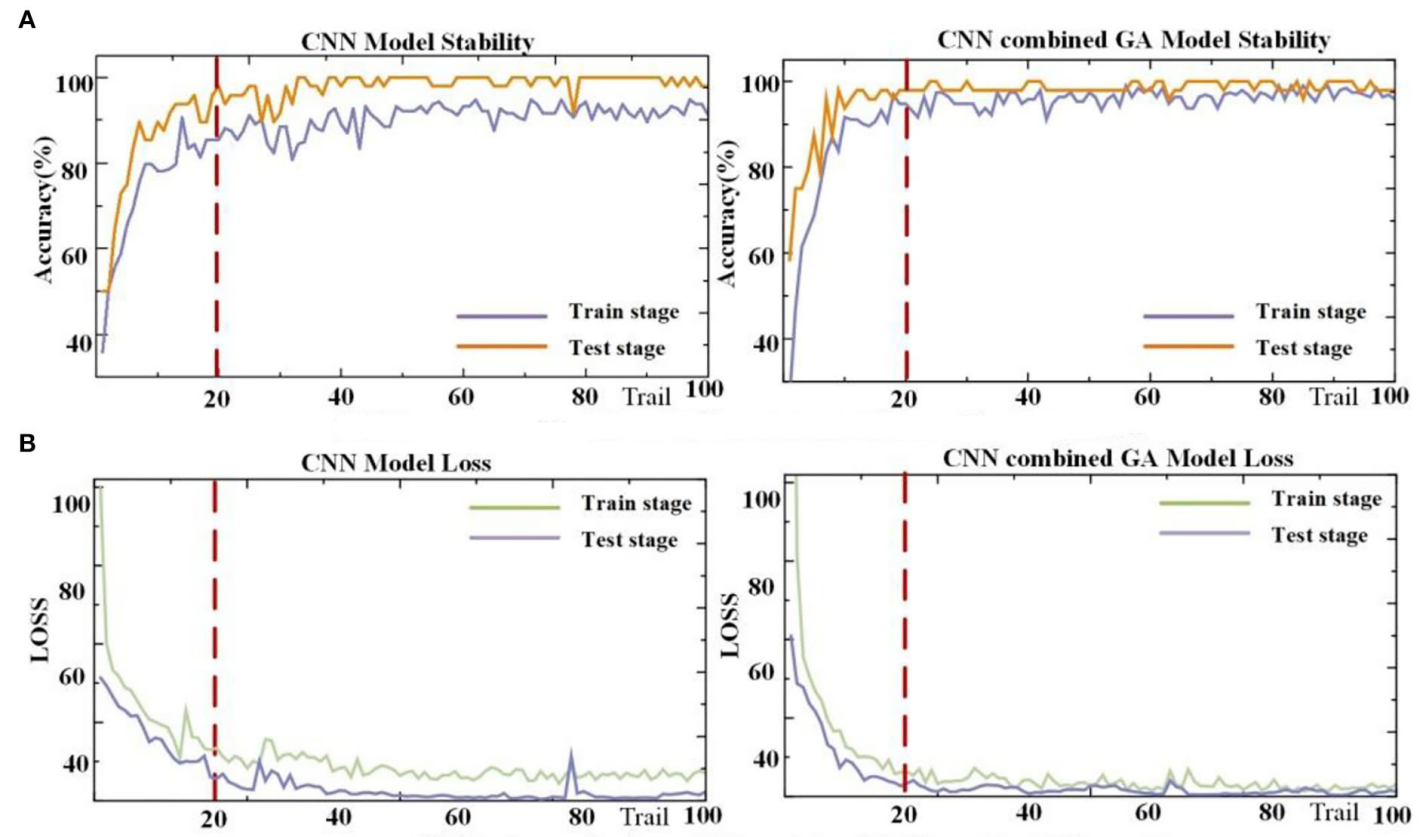
Performance from the CNN and CNN–GA algorithms. **(A)** The accuracy result from CNN model and CNN combined GA model. **(B)** The loss value from CNN model and CNN combined GA model.

Before verifying the effectiveness of the CNN–GA method for all subjects, data from one representative subject, S2, were thoroughly analyzed. Other participants reported similar results. Figure 6A depicts the decoding trends of the CNN–GA classifier and CNN for S2. The two algorithms exhibited similar trends after 20 epochs. However, several differences were observed during the training and testing stages. Figure 6B compares the stability and model loss between the CNN and CNN–GA classifiers. The improved CNN–GA model outperformed the traditional CNN algorithm, and its predicted targets varied slightly in both the training and testing stages, with CNN–GA loss values (after 20 epochs) of 1.024 and 1.456, respectively. The loss values of the CNN model were comparatively higher (at 1.683 and 2.457, respectively) under the same conditions. The analyzed results indicated that the hyperparameter optimization strategy could significantly improve the CNN performance.

**Figure 7:**
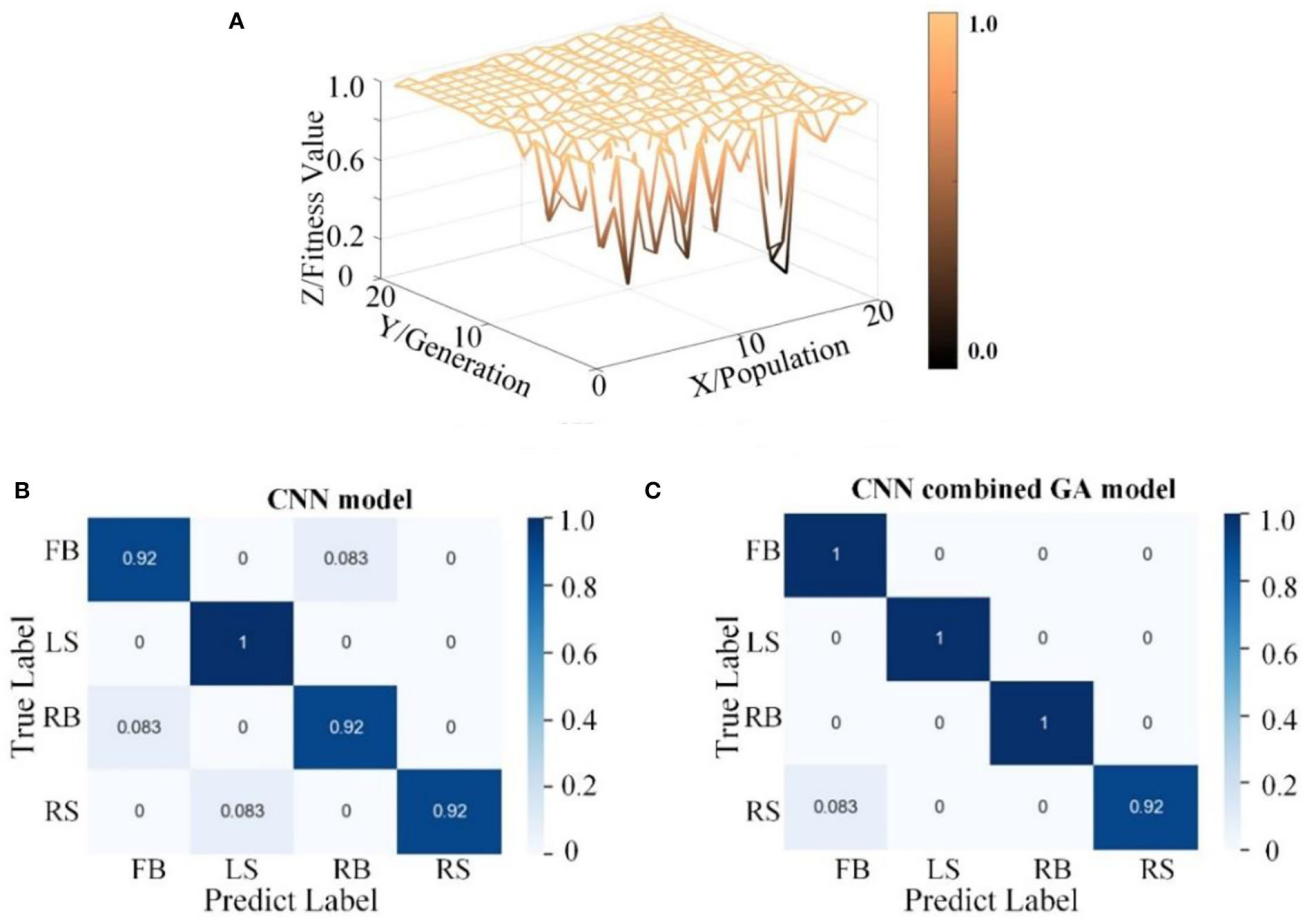
Hyperparameter optimization performance from the CNN combined GA. **(A)** The process of hyperparameter optimization by GA. **(B)** The confusion matrix from CNN and CNN combined GA.

To further analyze the performance of the CNN–GA algorithm in the FE-BCI system, the GA optimization process (with genetic offspring of size 20) and confusion matrices for CNN and CNN–GA were evaluated. Figure 7 shows the results for S2. Figure 7A depicts the process of hyperparameter optimization, where the *x*-axis indicates the population size, the *y*-axis indicates the generation size, and the *z*-axis indicates the accuracy across the different iterations. It is not difficult to find that the classification accuracy was gradually improved. After 15 iterations, the accuracy improved slightly, though the difference was not significant. This confirmed our previous hypothesis that it was feasible to improve the accuracy of the FE-BCI system using the GA optimization algorithm. The best hyperparameter optimization result for S2 is {15, 13, 6, 286, 68}. The first three numbers are the number of convolutional kernels, and the last two are the number of fully connected layer neurons.

As shown in Figure 7B, a confusion matrix was applied to demonstrate the superiority of the CNN–GA algorithm. The *x*- and *y*-axes denote the true and predicted values, respectively. Comparing the confusion matrices from the two methods, the overall accuracy was seen to be improved by 4%, which further demonstrates that the CNN–GA model can easily and accurately predict positive samples.

The performances obtained from the spatiotemporal analysis showed that the CNN–GA model efficiently distinguished the EEG signals from different facial expressions. Table 1 further analyzes the testing classification for S2. The average accuracy for S2 in the two-round five-fold cross-validation was 97.71 ± 2.07%. The highest accuracy was 100% and the lowest was 93.75%.

**Table 1:** Offline accuracies of the CNN–GA for S2.

	**1**	**2**	**3**	**4**	**5**	**6**	**7**	**8**	**9**	**10**	**Mean**
Acc (%)	97.92	100	97.92	95.83	93.75	95.83	97.92	100	100	97.92	97.71 ± 2.07

To compare the optimization performances of the hyperparameters, the averaged classification accuracy and its standard deviation with and without GA optimization are listed in Table 2. The average accuracies achieved by CNN and CNN–GA for all subjects were 85.94 ± 6.51 and 89.21 ± 3.79%, respectively. The averaged kappa value increased from 0.816 to 0.856. The highest recognition accuracy was obtained for S2 (up to 97.71 ± 2.07%); the lowest accuracy was obtained for S4 (76.43 ± 7.13%). The proposed algorithm improved the average accuracy by 3.27%, and the standard deviation was reduced by 2.72% for all subjects. The recognition accuracy for S11 was significantly increased from 89.27 ± 6.53% to 94.79 ± 3.54%; this increased the accuracy by 5.52% and decreased its standard deviation by 2.99%. The average classification accuracy across eight subjects exceeded 91.25%.

**Table 2:** Averaged accuracies for each subject under the CNN and CNN–GA methods.

**Subject**	**CNN**		**CNN–GA**	
	**Test (%)**	**Kappa**	**Test (%)**	**Kappa**
S1	84.90 ± 5.77	0.80	87.06 ± 4.51	0.83
S2	94.48 ± 3.90	0.93	97.71 ± 2.07	0.96
S3	80.52 ± 4.74	0.74	85.21 ± 4.92	0.80
S4	72.80 ± 6.22	0.61	76.43 ± 7.13	0.69
S5	88.75 ± 7.13	0.85	91.46 ± 5.27	0.89
S6	92.29 ± 6.93	0.90	96.88 ± 2.75	0.96
S7	86.77 ± 6.56	0.82	91.25 ± 2.75	0.88
S8	82.55 ± 6.22	0.77	86.09 ± 3.96	0.81
S9	83.13 ± 3.29	0.78	84.90 ± 2.27	0.80
S10	92.81 ± 16.29	0.92	95.83 ± 3.38	0.94
S11	89.27 ± 6.53	0.91	94.79 ± 3.54	0.93
S12	95.63 ± 6.34	0.94	96.77 ± 2.19	0.96
S13	78.54 ± 5.97	0.71	81.88 ± 3.39	0.76
S14	81.46 ± 6.08	0.75	83.52 ± 3.02	0.78
S15	81.15 ± 6.06	0.75	85.21 ± 4.87	0.80
S16	90.00 ± 6.07	0.87	93.02 ± 3.90	0.91
Avg ± Std	85.94 ± 6.51	0.816	89.21 ± 3.79	0.856

To statistically compare the performances of the two classifiers, a paired *t*-test was conducted. The results showed a considerable difference between the two algorithms (*p* < 0.05). This also suggests that the hyperparameter optimization method can effectively optimize the classifier performance. Despite these general experimental results, inter-subject variability still occurred. This phenomenon may have been caused by attention attenuation or mental fatigue during repetitive facial tasks.

To determine the efficiency of the selected parameter-optimization method in the FE-BCI system, the classification accuracies of the traditional method and our proposed method are also compared in Table 3. The average accuracy under the CNN–GA method was 89.21 ± 3.79%; meanwhile, the overall average accuracy of the traditional WT-BPNN method was 81.60 ± 7.36%. The average accuracy increased by 7.61%, while its standard deviation decreased by 3.57%. The recognition accuracy for S7 increased significantly from 79.17 ± 9.17 to 91.25 ± 2.75%; this increased the accuracy by 12.08% and reduced the standard deviation by 6.42%.

**Table 3:** Averaged accuracies of each subject under WT–BPNN and CNN–GA methods.

	**Accuracy (%)**			
**Subjects**	**WT–BPNN**		**CNN–GA**	
	**Kappa**	**Test (%)**	**Kappa**	**Test (%)**
S1	0.754	81.56 ± 6.77	0.828	87.06 ± 4.51
S2	0.874	90.42 ± 6.57	0.970	97.19 ± 2.81
S3	0.683	76.25 ± 8.18	0.803	85.21 ± 4.92
S4	0.613	70.94 ± 7.60	0.686	76.43 ± 7.13
S5	0.774	83.02 ± 8.64	0.886	91.46 ± 5.27
S6	0.879	90.94 ± 4.07	0.958	96.88 ± 2.75
S7	0.722	79.17 ± 9.17	0.883	91.25 ± 2.75
S8	0.739	80.42 ± 6.25	0.815	86.09 ± 3.96
S9	0.742	80.63 ± 6.04	0.799	84.90 ± 2.27
S10	0.803	85.21 ± 6.48	0.944	95.83 ± 3.38
S11	0.828	87.08 ± 8.78	0.931	94.79 ± 3.54
S12	0.833	87.50 ± 10.71	0.957	96.77 ± 2.19
S13	0.667	75.00 ± 7.34	0.758	81.88 ± 3.39
S14	0.690	76.77 ± 7.26	0.780	83.52 ± 3.02
S15	0.701	77.60 ± 7.26	0.803	85.21 ± 4.87
S16	0.776	83.23 ± 6.63	0.907	93.02 ± 3.90
Mean accuracy	0.755 ± 0.076	81.60 ± 7.36	0.857 ± 0.084	89.21 ± 3.79

Furthermore, a statistical analysis was conducted using a paired *t*-test, to investigate whether the two algorithms had any significant differences. The results indicated a significant difference between the two methods, and the accuracy of CNN–GA outperformed that of the combined WT–BPNN (*P* < 0.05). The experimental results showed that the proposed optimization algorithm achieved a higher classification rate and superior robustness for all subjects.

**Figure 8:**
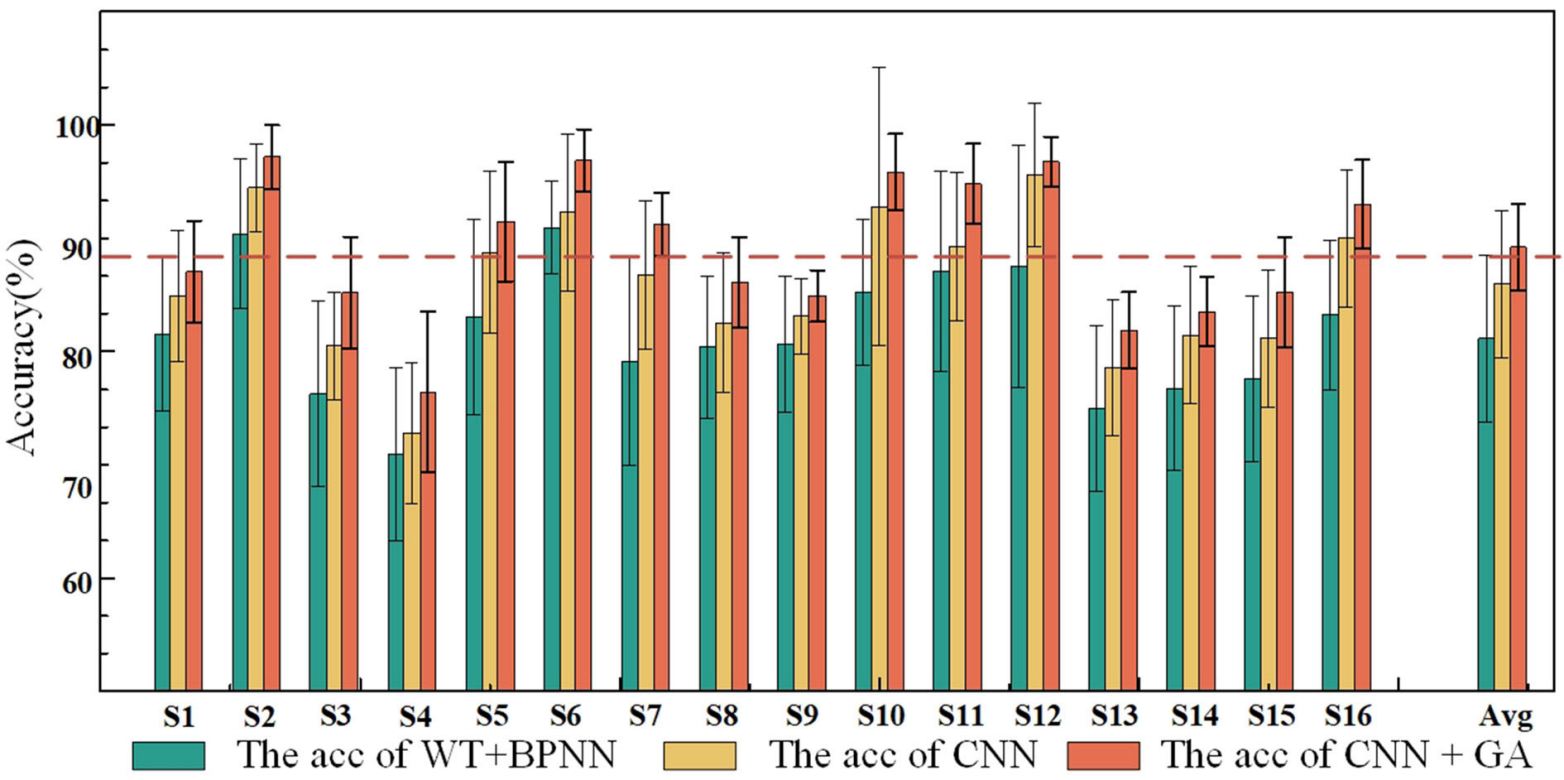
Offline classification accuracies and standard deviations under the three methods.

To further investigate the performance of the proposed method, the classification results achieved by the three different methods are compared in Figure 8. The results verified that the performance of the CNN–GA surpassed that of the other two methods. One-way ANOVA was used to assess the performances under the three conditions, and significant differences were observed among the three conditions (*P* < 0.05). The experimental results further validated the efficiency of the proposed method for detecting the characteristics of EEG signals produced by different facial expressions.

The above results demonstrate that the optimization method proposed in this study is effective for decoding EEG signals for FE-BCI systems.

### Online experimental results

**Figure 9:**
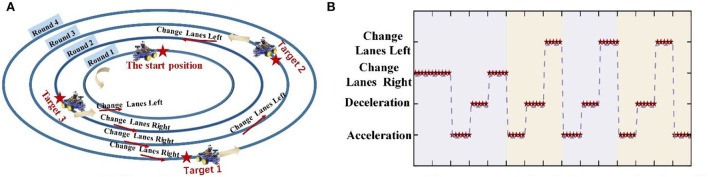
Online scenario and optimal recognition performance within a single session for S2. **(A)** The online scene. **(B)** One representative decision procedure from S2.

The offline experimental results demonstrated the feasibility of the FE-BCI system, and the online experiment was designed to verify the practicality of the optimized FE-BCI system used for vehicle control. To preliminarily evaluate the online performance of the FE-BCI system for controlling an intelligent car, the success rate was calculated. During the online experiment, all subjects could cross Targets 1 to 3 and then return to the start position. Each session included three left-hand lane changes, three right-hand lane changes, five accelerations, and four decelerations. In the control stage, subjects were required to maintain the same facial expression for 1.5 s to generate a car movement decision; for the online task, this time window was set to 0.25 s. The intelligent car remained in the previous state until the new control commands had been generated three times in the same. Figure 9 shows the experimental scenario and a representative decision procedure from S2.

The task success rates for each subject are listed in Table 4. The average success rate across six sessions was 86.61 ± 6.06%, and the highest was 96.31 ± 2.71% from S10. The mean standard deviation during the online experiment was 6.06%, which further suggests the robustness of the proposed GA–CNN in applications.

**Table 4:** Averaged accuracies of each subject in the online task.

**Subjects**	**Acc. (%)**				
	**Change left**	**Acceleration**	**Change right**	**Deceleration**	**Mean**
S1	79.41	83.33	73.33	88.89	81.24 ± 6.55
S2	97.06	88.89	96.67	100	95.65 ± 4.75
S3	70.59	77.78	100	94.44	85.70 ± 13.81
S4	76.47	77.78	83.33	77.78	78.84 ± 3.06
S5	91.18	83.33	93.33	94.44	90.57 ± 5.01
S6	94.12	100	96.67	94.44	96.31 ± 2.71
S7	94.17	88.89	90.00	77.78	85.56 ± 6.76
S8	97.06	88.89	93.33	94.44	93.43 ± 3.41
S9	85.29	83.33	83.33	72.22	81.04 ± 5.95
S10	94.12	100	96.67	94.44	96.31 ± 2.71
S11	91.17	83.33	76.67	88.89	85.01 ± 6.46
S12	97.06	88.89	93.33	88.89	92.04 ± 3.95
S13	79.41	88.89	83.33	83.33	83.74 ± 3.90
S14	85.29	77.78	83.33	77.78	81.05 ± 3.85
S15	82.35	83.33	90.00	77.78	83.36 ± 5.04
S16	85.29	77.78	80.00	72.22	78.82 ± 5.41
Mean ± Std	87.50 ± 8.22	85.41 ± 6.37	88.33 ± 7.98	85.76 ± 8.83	86.61 ± 6.06

## Discussion

Existing disparities between the selectivity of BCI systems and their performance mean that there is plenty of room for improvement in current BCI systems. In this study, a novel FE-BCI system with an improved EEG decoding method combining a CNN with GA was proposed. The offline experimental results demonstrated that the improved deep learning method was able to significantly improve the model performance compared to the traditional method. The online experimental results verified the feasibility of the proposed FE-BCI system for practical applications. Notably, the improved FE-BCI system outperformed the conventional BCI system.

### Significance of the FE-BCI system

Emotion computation plays an important role in human communication and real-world applications. Although effective computation has attracted considerable interest in the past few years, the use of emotions in brain-controlled systems remains problematic. Research in the affective BCI field has focused on perception; numerous specific stimuli have been used to detect different emotions (e.g., emotional videos, images, and music). According to Jiang et al. different emotions can be evoked by a video's affective content and further applied in an emotion-based BCI system (Jiang et al., 2019).

Jin and his colleagues reported on a new emotion-detecting BCI system that employs a face-based image-induced paradigm (Cheng et al., 2017). In another study, Thammasan et al. studied a continuous music-emotion-recognition approach for the construction of affective BCI (Thammasan et al., 2016). However, it is difficult to detect the ground truth of human emotional states using these methods. Most importantly, these paradigms rely upon extra stimuli, which limits their real-world applications.

In contrast to traditional emotion discrimination techniques, it is more straightforward to recognize the emotional stage using different facial expressions. Facial expressions are the most common features of emotions and the most direct mechanism of emotional representation. Unfortunately, the inconsistency of emotion and expression still has been the main challenge for this type of emotion-based BCI system. Since the facial expression is a kind of body movement, the combination of EEG signals from the prefrontal and motor cortices could improve the robustness and credibility of techniques that exploit these signals. This way could also reduce the impact on the decoding accuracy of FE-BCI signals when expressions are inconsistent with emotions.

In our approach, EEG signals from the prefrontal and motor cortices were used to distinguish between different emotional states. Furthermore, a highly robust EEG recognition model was obtained by combining a CNN with a GA. To prove the effectiveness of the proposed FE-BCI system, Table 5 shows the results of representative EEG decoding algorithms for expression-based brain-computer interfaces system in the past few years. It can be seen that the decoding accuracy of the P300-based visually evoked BCI system was still relatively high (Cheng et al., 2017; Tian et al., 2018). The performance of this type of the FE-BCI system depends entirely on the design of the stimulator, which includes the size of the face picture, the space between two pictures, and the number of target appearances. In contrast, the method proposed in this study shows the superiority in the film video elicitation with WT- MLPNN and STFT combined Graph Regularized Extreme Learning Machine (Ozerdem and Polat, 2017; Zheng et al., 2019), music elicitation with Higuchi algorithm combined SVM (Thammasan et al., 2016), the pictures of facial expression elicitation (Huang et al., 2017) with mixed features and corresponding algorithms, and the previously proposed actual facial expression-based WT-BPNN decoding method (Toth and Arvaneh, 2017; Li et al., 2018b). This improvement will further extend the broader range of human-computer interaction. In contrast to previous studies, we used only eight-channel EEG signals, and the average accuracy was as high as 89.21 ± 3.79%. This will further extend the possibilities of human–computer interaction.

**Table 5:** Performance comparison of previous work based on the FE-BCI.

**References**	**Modality**	**Stimulus**	**Method**	**Parameters**	**Acc (%)**
Cheng et al. (2017)	EEG (P300)	P 300 evoked	Features extracted by calculating the percentiles of EEG; Classified by Bayesian linear discriminant analysis	Referring previous study	91.9
Tian et al. (2018)	EEG(N170)		N170 extracted by dimensionality reduction and normalization; Classified by L1-Regularized Logistic Regression		86.4
Thammasan et al. (2016)	EEG	Music	Features extracted by Higuchi algorithm; Classified by SVM	By experience	85.0
Ozerdem and Polat (2017)	EEG	Film chips	Features extracted by wavelet transform; classified by MLPNN	Referring previous study	77.14
Zheng et al. (2019)			Features extracted by STFT; classified by Graph Regularized Extreme Learning Machine		69.67
Huang et al. (2017)	• Picture information • EEG	• Face pictures • Facial expression	• Picture feature extracted by AdaBoost and classified by neural network classifier • EEG feature extracted by STFT and classified by SVM	Burte-Force Searching	82.75
Toth and Arvaneh (2017)	• EEG • Gyroscope	Facial expression	Feature extracted by FFT; classified by SVM-LDA-Bayesian	By experience	70.3
Li et al. (2018b)	EEG		Features extracted by wavelet transform; classified by BPNN		81.28
The proposed study			Features extracted and classified by CNN	By GA	89.21

### Efficacy of CNN–GA

Owing to the inherent signal quality limitations of non-invasive EEG signals, there remains a need to develop a novel EEG decoding algorithm that improves the precision of facial-expression-based BCI systems. Most conventional machine learning algorithms set these features manually; thus, they are highly dependent upon the experience of the researcher. However, irrelevant features reduce the classifier performance. Hence, selecting features relevant to the task will improve the classification performance. One advantage of CNN is the automatic extraction of discriminative features (Shajil et al., 2020; Kwon and Im, 2021). Learning hidden features and eliminating redundant information from the EEG signals will enhance the overall capability of BCI systems. Using the classification accuracy metric, Tables 3, 5 present a comparison of the improved CNN and traditional methods. The proposed scheme outperformed other traditional feature extraction and classification methods. Hence, automatic learning of relevant features and eliminating redundant information from EEG signals could effectively improve the recognition accuracy of EEG signals under different expressing.

It is well-known that the performance of a neural network model is highly dependent upon its hyperparameters (Ali et al., 2019). However, most hyperparameter optimizations use the enumeration method to solve this problem. Unfortunately, the selection of inappropriate hyperparameters may result in a poor classification performance. In contrast to selecting hyperparameters using research experience, the proposed scheme sets important hyperparameters using the GA algorithm. GA has an excellent global search ability, which can quickly search out the best solution in the solution space without any prior knowledge. The GA's paralleling process uses numerous routes to find the optimal results. This characteristics ensures that the best solution was found while avoiding fast-falling trap of the optimal local solution (Rui et al., 2019). Most important, the superior performance of the GA method is its social ability, which makes it easier to link with other algorithms (Chang and Yang, 2019). Therefore, embedding the GA algorithm into the CNN model by setting this model as a fitness function is an effective way to optimize the decoding results of EEG signals. Table 2 presents a systematic comparison and qualitative evaluation of the proposed model with and without hyperparameter optimization. Superior accuracy was achieved compared to the CNN model. This experimental result verified that the GA-optimized hyperparameters improved the classification performance and further resolved the time-consumption problem of redundant information. These analytic results demonstrate that the CNN–GA EEG decoding model can produce a more interpretable model for exploring the information hidden in EEG signals. This should promote the development of a high-quality EEG decoding model.

### Comparison with existing BCI systems

The practical performance of BCI systems is worth discussing. Existing challenges to the practical implementation of BCI systems include their accuracy, portability, and robustness. The MI-based BCI system is a representative BCI system used to improve the quality of life of disabled people. For example, Miao reported an MI-BCI system that helped stroke patients toward rehabilitation (Miao et al., 2021). However, this type of BCI system does not readily facilitate daily activities, owing to its low accuracy and limited commands. Recently, a great surge in SSVEP-based BCI systems has been observed in daily life applications. For example, Chen et al. produced a robotic arm control mechanism using an SSVEP system (Chen et al., 2021). Unfortunately, the performances of most existing strategies are highly dependent upon extra stimuli. This partially limits the mobility of the SSVEP–BCI system.

The development of the FE-BCI system provides an additional option for solving the tradeoff between BCI performance and stimulus reliance. Compared with the traditional BCI system, the EEG signals from real facial expressions can increase the portability of the FE-BCI system. EEG decoding algorithms also play a vital role in BCI systems. In this study, the combined CNN–GA model also ensured the accuracy of the FE-BCI system. Hence, the improved FE-BCI system is cost-effective, user-convenient, and more suitable for practical tasks.

### Limitations and future work

Despite the superior performance of the improved FE-BCI system, certain aspects still need to be improved. One limitation of this study is that only healthy subjects were considered, and a relatively small number of subjects participated. In future studies, clinical applications involving certain patient groups and more subjects should be included. Moreover, enhancing the generalizability of the classifier and studying the asynchronous FE-BCI system should produce a better solution and provide a more flexible and realistic BCI system. There remains the motivation for finding more computationally and friendly metrics to investigate the consistency between emotions and expressions. Exploring a more advanced algorithm and effective criteria to reduce inter-subject variability will remain a challenge to be addressed in the future.

## Conclusion

This paper proposed a novel deep-learning-based EEG decoding method for an FE-BCI system, and the performance of the proposed CNN–GA model was evaluated systematically. The proposed method employed a CNN algorithm to decode EEG signals and a GA to select the optimal hyperparameters for the CNN model. To verify the model effectiveness, offline and online experiments were conducted. When using the CNN–GA algorithm in offline experiments, the averaged accuracies were increased from 85.94 ± 6.51 to 81.60 ± 7.36% (for the conventional CNN algorithm and traditional BPNN-based method, respectively) to 89.21 ± 3.79%. Moreover, the online experiment results demonstrated the practical applicability of the method, and the average accuracy was increased up to 86.61 ± 6.06%. Both the offline and online experimental results demonstrated the superiority of the proposed EEG decoding method in the FE-BCI system. In summary, the CNN–GA method is a significant achievement in the development of FE-BCI systems. Future work will aim to develop an asynchronous FE-BCI system using the CNN–GA model; this will further improve paralyzed patients' access to the real world.

## Data availability statement

The raw data supporting the conclusions of this article will be made available by the authors, without undue reservation.

## Ethics statement

The studies involving human participants were reviewed and approved by the Institutional Review Board of Xi'an University of Technology. All experiments were conducted in accordance with the of Helsinki. The patients/participants provided their written informed consent to participate in this study. Written informed consent was obtained from the individual(s) for the publication of any potentially identifiable images or data included in this article.

## Author contributions

RL and DL organized the experiments and wrote the manuscript. ZL and JL did the research and revised the manuscript. WF and WL supervised the work and carried out the experiments. BL analyzed the data. JZ and AA modified the grammar of this article. All authors contributed to the article and approved the submitted version.

## Funding

This research work was supported by the Natural Science Foundation of Shaanxi (Grant No. 2022JQ-402) and National Defense Science and Technology Foundation Strengthening Program Technology Field Fund Project (Grant No. 2020-JCJQ-JJ-367).

## Conflict of interest

The authors declare that the research was conducted in the absence of any commercial or financial relationships that could be construed as a potential conflict of interest.

## Publisher's note

All claims expressed in this article are solely those of the authors and do not necessarily represent those of their affiliated organizations, or those of the publisher, the editors and the reviewers. Any product that may be evaluated in this article, or claim that may be made by its manufacturer, is not guaranteed or endorsed by the publisher.
